# Opsoclonus myoclonus ataxia may differentiate postinfectious autoimmune encephalitis from infectious encephalitis

**DOI:** 10.1007/s10072-021-05632-1

**Published:** 2021-09-29

**Authors:** Seong-Joon Lee, In Ja Shin, Tae-Joon Kim

**Affiliations:** Department of Neurology, Ajou University School of Medicine, Ajou University Medical Center, 164, World cup-ro, Yeongtong-gu, Suwon, Gyeonggi-do 16499 Republic of Korea

Dear Editor,

We report a patient who could be diagnosed as autoimmune postinfectious encephalitis due to occurrence of opsoclonus myoclonus ataxia syndrome (OMS) while initially treated under the impression of herpes simplex virus (HSV) encephalitis.

A 19-year-old male presented with 4 days of fever, headache, and nausea. Upon clinical examination, meningeal irritation sign was positive without focal neurological deficits. Cerebrospinal fluid (CSF) study showed elevated opening pressure, white blood cell count of 130 (lymphocyte 86%), elevated protein of 154 mg/dL, and glucose of 50 mg/dL. Brain magnetic resonance imaging (MRI) showed T2 hyperintensities and swelling of bilateral medial temporal lobes (Fig. 1A). Serologic test for HSV IgM was equivocal, while *Mycoplasma pneumoniae* IgM was positive (titer 2.6 [0.0–0.8]). CSF HSV PCR was negative (Table 1). Under the impression of HSV meningoencephalitis, IV acyclovir and oral doxycycline was started. On HOD2 patient developed diplopia, downbeat, and torsional nystagmus. Over the next few days, the patient was neurologically stable, but fever did not subside. The patient deteriorated on HOD 6 with fluctuating right ptosis and esotropia. Intermittent bouts of uncontrolled multivectorial rapid eye movements which could be classified as opsoclonus occurred (Fig. 2, Video) with truncal ataxia and limb dysmetria. The frequency of opsoclonus was 6 ~ 8 Hz, with an amplitude of 5 ~ 10 degrees, and was provoked by initiation of saccades. The patient also complained of confusion and vivid dreams. Under the syndromatic diagnosis of OMS, postinfectious etiology was suspected, and IV steroid therapy was started. The results of paraneoplastic antibodies and autoimmune synaptic antibodies were negative. There was partial response to steroids, but the patient complained of urinary retention. Brain MRI on HOD12 revealed patchy T2 high signal intensities in cerebellar dentate nucleus, bilateral thalamus, and basal ganglia (Fig. 1A). Spinal MRI revealed signal changes in T1–4 and T4–T12 levels (Fig. 1B). The patient further underwent 5 cycles of plasma exchange with excellent clinical response. Serologic exam at HOD17 showed negative conversion of HSV IgM and positive HSV IgG. *Mycoplasma pneumoniae* IgM also showed negative conversion, with positive *Mycoplasma pneumoniae* IgG (> 100AU/mL [0.0–11.9]). The patient was able to be discharged home at HOD24 and has not shown recurrence for over 6 months.

**Fig. 1:**
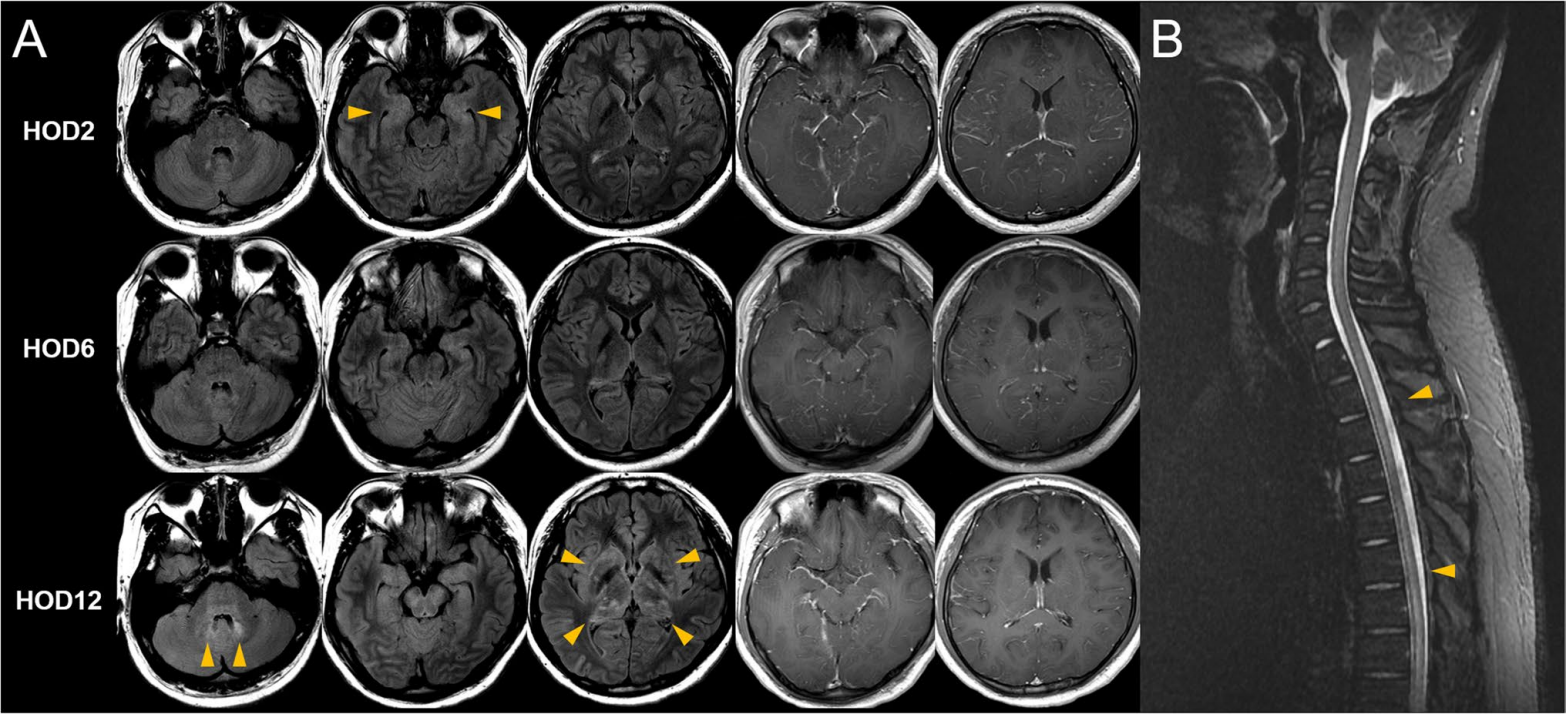
**The brain and spinal MRI findings.** **A** On HOD2, swelling and T2 high signal intensity are seen at bilateral medial temporal lobes, raising clinical suspicion of herpes simplex encephalitis. The patient deteriorated on HOD6, but no new brain lesions are seen. Brain MRI performed at HOD12 shows T2 hyperintensities at cerebellar dentate nucleus, bilateral thalamus, and basal ganglia. **B** T2 hyperintensities in T1–4 and T4–T12 levels are also seen in spinal MRI taken at HOD12. MRI, magnetic resonance imaging; HOD, hospital day

**Table 1:** Results of serologic and cerebrospinal fluid exams for diagnosis of the encephalitis

	**HOD 1**	**HOD 6**	**HOD 8**	**HOD 13**	**HOD 15**	**HOD 17**
Serum tests						
CMV IgM	Negative					
HSV IgM	Equivocal					Negative
HSV IgG						Positive
VZV IgM	Negative					Negative
*M. pneumonia* IgM	Positive 2.6 (0.0–0.8)					Negative
*M. pneumoniae* IgG						Positive > 100AU/mL (0.0–11.9)
Anti-HIV I/II	Negative					
VDRL	Negative					
Paraneoplastic antibody*			Negative			
Autoimmune synaptic encephalitis Ab †						
GAD II Ab			Negative			
Aquaporin 4 IgG				Negative		
GD1b Ab, IgM				Negative		
GQ1b Ab				Negative		
MOG Ab				Negative		
Cerebrospinal fluid						
HSV type I PCR	Negative	Negative	Negative		Negative	
HSV type II PCR	Negative	Negative	Negative		Negative	
HSV RT PCR	Negative	Negative	Negative		Negative	
VZV PCR	Negative	Negative	Negative		Negative	
Enterovirus RT PCR	Negative					
EBV PCR					Negative	
HHV 6 PSR					Negative	
Polyoma Virus PCR					Negative	
*M. pneumoniae* PCR					Negative	
JBE virus RT PCR					Negative	
CMB RT PCR					Negative	
TB PCR hybridization	Negative					
VDRL				Negative		

^*^Paraneoplastic antibody testing was performed for anti-Hu autoantibody, anti-Ri autoantibody, anti-Yo autoantibody, anti-amphiphysin autoantibody, anti-CV2 autoantibody, anti-PNMA (Ma2/Ta) autoantibody, anti-recoverin autoantibody, anti-SOX1 autoantibody, and anti-titin (MGT-30) autoantibody

^†^Autoimmune synaptic encephalitis antibody was performed for anti-NMDA receptor antibody, anti-LGI1antibody, anti-CASPR2 antibody, anti-AMPA receptor antibody, anti-DPPX antibody, and anti-GABA-B receptor antibody

*CMV* cytomegalovirus, *HSV* herpes simplex virus, *HIV* human immunodeficiency virus, *VDRL* Venereal Disease Research Laboratory, *GAD* glutamic acid decarboxylase, *MOG* myelin oligodendrocyte glycoprotein, *VZV* Varicella zoster virus, *PCR* polymerase chain reaction, *RT PCR* real-time polymerase chain reaction, *EBV* Epstein-Barr virus, *HHV* human herpes virus, *JBE* Japanese B encephalitis, *TB Mycobacterium tuberculosis*

**Fig. 2:**
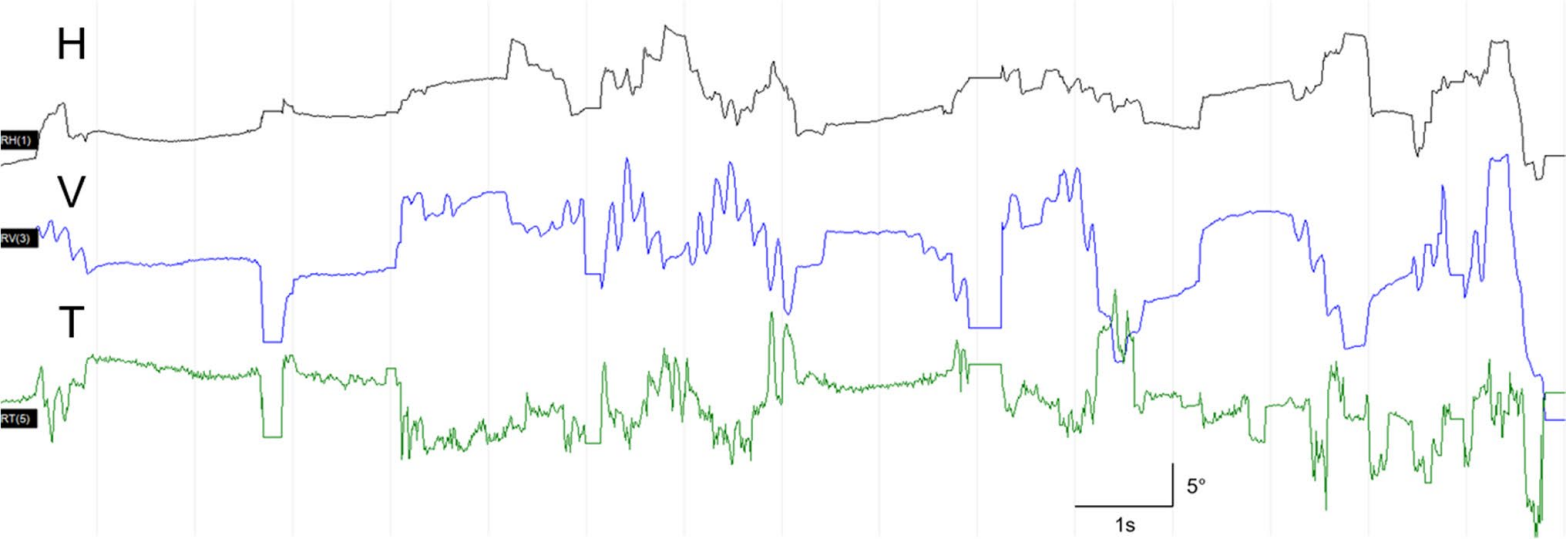
**VNG findings showing opsoclonus.** VNG (SLVNG, SLMED, Seoul, South Korea) shows intermittent bouts of fast involuntary spindle shaped multivectorial saccadic oscillations without intersaccadic interval. VNG, video nystagmography

The causes of OMS in adults include paraneoplastic, parainfectious, toxic-metabolic, and idiopathic etiologies, while the pathophysiology is thought to be immunological [7]. Reported parainfectious cases are associated with HIV, *Mycoplasma pneumoniae*, and *Salmonella*, among others [7]. On the contrary, neuro-ophthalmologic presentation in HSV encephalitis, other than cranial nerve signs, due to increased intracranial pressure, is rare [2, 8]. Based on such factors, the development of OMS led us to suspect an underlying autoimmune mechanism.

The preceding infection may have been HSV meningoencephalitis, or *Mycoplasma pneumoniae* infection. Reported clinical picture of post HSV autoimmune encephalitis is somewhat different from this patient [1], with longer days from initial encephalitis to relapsing autoimmune encephalitis, and neuropsychiatric presentations. Typical enhancing frontotemporal T2 hyperintensities and positive NMDA receptor antibodies were also not seen in this patient. Neurological manifestations of *Mycoplasma* infections occur through three major mechanisms, direct neuronal damage, vascular occlusion type pathology, and indirect autoimmunity [5]. Immune pathogenesis is usually suggested in cases of OMS associated with *Mycoplasma pneumoniae* [3, 4, 6]. Characteristic bilateral MRI lesions are reported, involving the pons, thalamus, basal ganglia, brainstem, or splenium, and there is evidence that both vascular occlusive pathology and indirect autoimmune mechanisms are responsible [5]. While we can not reach a conclusion to the underlying infectious pathogen, this case highlights that prompt recognition of OMS and immunomodulatory treatment can bring excellent outcomes.

## Supplementary Information

Below is the link to the electronic supplementary material.

Supplementary file1 (AVI 6977 KB)
